# Comparing the performance of dynamic susceptibility contrast and arterial spin labeling for detecting residual and recurrent glioblastoma with deep learning and multishell diffusion MRI

**DOI:** 10.1093/noajnl/vdaf219

**Published:** 2025-10-17

**Authors:** Louis Gagnon, Diviya Gupta, George Mastorakos, Nathan White, Vanessa Goodwill, Carrie R McDonald, Thomas Beaumont, Christopher Conlin, Tyler M Seibert, Uyen Nguyen, Jona Hattangadi-Gluth, Santosh Kesari, Jessica D Schulte, David Piccioni, Divya S Bolar, Anders M Dale, Nikdokht Farid, Jeffrey D Rudie

**Affiliations:** Department of Radiology, University of California San Diego, San Diego; Department of Radiology and Nuclear Medicine & CERVO Brain Research Center, Laval University, Quebec City; Department of Radiology, University of California San Diego, San Diego; Cortechs.ai, San Diego; Cortechs.ai, San Diego; Department of Pathology, University of California San Diego, San Diego; Department of Radiation Medicine and Applied ­Sciences, University of California San Diego, San Diego; Department of Neurological ­Surgery, University of California San Diego, San Diego; Department of Radiology, University of California San Diego, San Diego; Department of Radiology, University of California San Diego, San Diego; Department of Radiation Medicine and Applied ­Sciences, University of California San Diego, San Diego; Department of Bioengineering, University of ­California San Diego, San Diego; Department of Radiology, University of California San Diego, San Diego; Department of Radiation Medicine and Applied ­Sciences, University of California San Diego, San Diego; Department of Translational Neurosciences, Pacific Neuroscience Institute and Saint John’s Cancer Institute at Providence Saint Johns’ Health Center, Santa Monica; Department of Neurosciences, University of California San Diego, San Diego; Department of Neurosciences, University of California San Diego, San Diego; Department of Radiology, University of California San Diego, San Diego; Department of Radiology, University of California San Diego, San Diego; Department of Neurosciences, University of California San Diego, San Diego; Department of Radiology, University of California San Diego, San Diego; Department of Radiology, University of California San Diego, San Diego

**Keywords:** arterial spin labelingcellular tumordeep learning segmentationdynamic susceptibility contrastglioblastoma

## Abstract

**Background:**

Differentiating recurrent tumor from post-treatment changes remains a major challenge in glioblastoma (GBM) patients. In this work, we compared the performance of 2 different MR perfusion techniques, dynamic susceptibility contrast (DSC), and arterial spin labeling (ASL) to differentiate recurrent tumor and post-treatment changes from the volume of cellular tumor segmented from combined Deep Learning and multimodal MRI measurements, including multishell diffusion and perfusion.

**Methods:**

In this retrospective study, 137 MRIs from 107 patients with GBM were analyzed. Cellular tumor maps were segmented by 2 radiologists based on imaging, clinical history, and pathology. Multimodal MRI with perfusion and multishell diffusion were inputted into 5 nnU-Net Deep Learning models using either DSC or ASL with combination of multishell diffusion and standard MRI sequences to segment cellular tumor. Models with DSC and ASL were compared using segmentation performance (Dice score) and accuracy to detect recurrent tumor from post-treatment changes (area under the curve [AUC] under the receiver operating characteristic curve).

**Results:**

Segmentation performances were similar in both cases, with a median Dice score of 0.75 (IQR: 0.53-0.84) for ASL and 0.76 (IQR: 0.57-0.84). AUC was 0.88 (CI 0.82-0.94) for ASL and 0.86 (CI, 0.80-0.92) for DSC, and this difference was statistically significant (*P* < .05, *n* = 10 000 permutation test). In 11 individual cases, recurring disease was detected with ASL but missed with cerebral blood volume, including recurring tumor in the vicinity of a surgical cavity (*n* = 5), close to the skull base (*n* = 1), and adjacent to an Ommaya reservoir (*n* = 2).

**Conclusions:**

Our results demonstrate the utility of ASL in regions where susceptibility artifacts decrease the quality of DSC images.

Key Points

- Multishell dMRI measurements and Deep Learning can differentiate recurring GBM from post-treatment changes.
- Including an MR perfusion measurement as an additional input to the model increased the performance.
- ASL performs better in regions prone to susceptibility artifacts.

Importance of the study

Distinguishing recurring glioblastoma from post-treatment changes on brain MRI remains a challenge clinically. The combination of multishell diffusion MRI measurements and Deep Learning segmentation tools can accurately segment enhancing and non-enhancing cellular tumor, which can be used to distinguish recurrent/residual tumor from post-treatment changes. While it is known that including a perfusion sequence can improve the method, no direct comparison of different perfusion techniques has been performed. Here, we report an extensive comparison of 2 widely used MR perfusion methods—dynamic susceptibility contrast (DSC) and arterial spin labeling (ASL)—to segment enhancing and non-enhancing tumor and detect recurring disease. We demonstrate that ASL performs better in specific instances where susceptibility artifacts can decrease the quality of DSC—including in the periphery of a surgical cavity with blood products, close to the skull base, and adjacent to an Ommaya reservoir—emphasizing the benefits of including ASL in brain tumor protocols.

Glioblastoma (GBM) is the most aggressive and most common type of cancer that originates in the brain and has a very poor prognosis for survival of 15-21 months post-diagnosis.^1^^,^^2^ Multimodal MRI plays a crucial role in assessing tumor burden, guiding surgical planning, and monitoring disease progression both pre- and post-surgery. However, differentiating recurrent tumor from post-treatment changes remains a significant challenge.^3^ Perfusion-weighted MRI techniques have demonstrated potential in distinguishing recurrent disease from post-treatment changes. High-grade gliomas, such as GBM, exhibit significant microvascular proliferation and pronounced angiogenesis,^4–6^ whereas pseudoprogression (PsP) and radiation necrosis are associated with small vessel damage and ischemia.^7^ Leveraging these physiological differences, perfusion measurements have been shown to accurately differentiate recurrent GBM from radiation necrosis.^8–10^

A common MR perfusion technique is dynamic susceptibility contrast (DSC), which estimates tissue microvascular density by measuring cerebral blood volume (CBV) and cerebral blood flow (CBF).^11^^,^^12^ However, DSC-MRI has several limitations. It relies on gadolinium-based contrast agents (GBCAs), some of which have been linked to nephrogenic systemic fibrosis in patients with impaired renal function.^13^ Additionally, all GBCAs accumulate in various tissues, including the brain, regardless of renal function.^14^ A well-known issue with DSC-MRI is contrast leakage into the extravascular space in areas with blood-brain barrier disruption (commonly seen in enhancing tumors), leading to T1-weighted (T1)-relaxation effects that can underestimate true CBV. Although various correction algorithms exist, each has its advantages and disadvantages, and there is no consensus on the optimal approach.^15–17^ Moreover, variations in processing techniques result in heterogeneous cutoff values, limiting comparability across studies.^16^^,^^18^ Another drawback of DSC-MRI is its reliance on gradient-echo sequences, which are susceptible to artifacts that can obscure lesion evaluation, particularly along the skull base, paranasal sinuses, and resection cavity margins, as well as in regions with hemorrhage.^16^

Arterial spin labeling (ASL) is an alternative, non-contrast perfusion-weighted MRI technique that is gaining recognition and may offer advantages over GBCA-based perfusion imaging. ASL uses arterial blood as an endogenous tracer by magnetically labeling inflowing blood with radiofrequency inversion pulses, enabling absolute tissue perfusion measurements.^19^^,^^20^ Recent advancements, particularly the adoption of the white paper-recommended three-dimensional pseudocontinuous ASL (3D PCASL) sequence,^21^ have contributed to ASL’s increasing popularity for perfusion imaging. ASL has shown promise in neuro-oncologic applications, including the assessment of brain tumor perfusion^22–24^ and differentiation between low- and high-grade gliomas.^25–27^ Several studies have explored ASL’s potential for distinguishing recurrent tumor from treatment effects, with early findings suggesting that ASL may serve as an alternative perfusion MRI technique for this purpose.^27–33^

Recently, we have shown that the combination of multishell diffusion MRI measurements and Deep Learning segmentation tools can accurately segment enhancing and non-enhancing cellular tumor (NECT), classify recurrent/residual tumor from post-treatment changes, and predict Overall Survival and Progression-Free Survival^34^ among GBM patients. While our previous work showed that including an MR perfusion measurement as an additional input to the model increased performance, the effect of the type of perfusion used on the performance of the method has not been studied. In this work, we compare the performance of DSC and ASL to segment enhancing and NECT and to detect recurring disease from the volume of segmented cellular tumor.

## Methods

### Study Design and Patients

**Figure 1.**
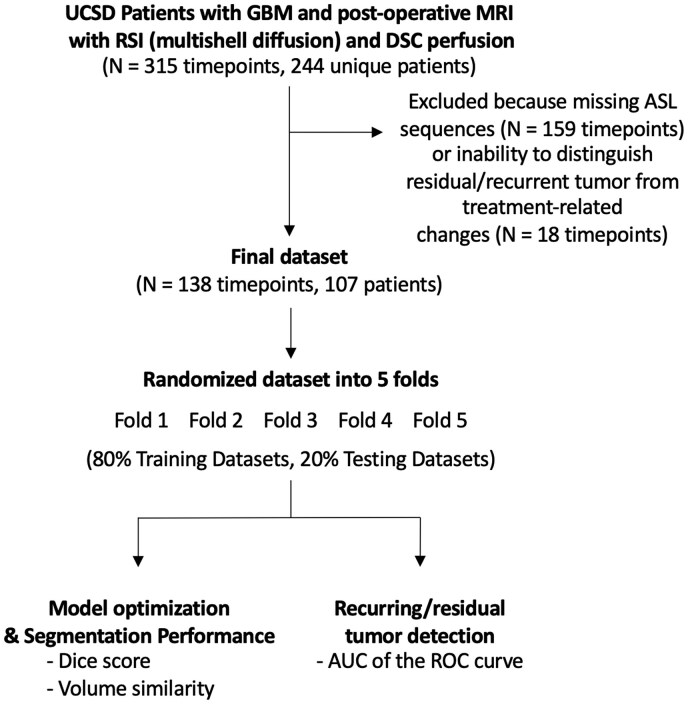
Overview of the experimental design of the study. A 5-fold analysis was performed for model optimization and assessing model segmentation performance, and in order to quantify the performance of the model to detect residual/recurring tumor.

Figure 1 outlines the study design. Table S1 presents patient demographics. This retrospective study received approval from the institutional review board of the University of California San Diego (UCSD), and the requirement for informed consent was waived. Data were de-identified and collected according to the Health Insurance Portability and Accountability Act (HIPAA) standard. A portion (*n* = 137/243) of this dataset overlaps with our previous work^34^ with the addition of ASL data. Patients diagnosed with GBM, following the fourth edition (2016) of the WHO classification, were identified through a chart review of the institutional medical records (3M M×Modal Scout) between January 2018 and June 2022 at UCSD. Eligible patients included those who had post-treatment MRIs with multishell diffusion sequence Restricted Spectrum Imaging (RSI) and ASL. To enhance the dataset, the pathology database was queried for cases with pathology-confirmed progression and treatment-related changes. Initially, 244 unique patients (315 timepoints) were identified, but 159 timepoints were excluded due to missing ASL sequences, and 18 were rejected because it was not possible to confidently differentiate between treatment-related changes and residual or recurrent tumors (Figure 1). The final cohort consisted of 107 patients (138 timepoints), with a mean age of 57 ± 13 years (SD), comprising 70 men and 37 women. Among the timepoints, 112 showed residual or recurrent tumors, while 26 exhibited only post-treatment changes. Clinically confirmed PsP was defined as an increase in the size of an enhancing component within 0-6 months after combined chemotherapy and radiotherapy, followed by a spontaneous decrease in size on subsequent follow-ups without further treatment. Radiation necrosis was identified as an increase in the size of a ring-enhancing lesion with a necrotic core following radiotherapy, either confirmed through pathology or identified by neuroradiologists. Non-specific post-treatment changes were classified as areas of abnormal enhancement or T2-weighted (T2)/fluid-attenuated inversion recovery (FLAIR) hyperintensity related to treatment that did not meet the criteria for PsP, radiation necrosis, or recurrent disease.

### Data Acquisition

Each patient underwent imaging using a standard brain tumor protocol that included the following sequences: T1, T1-weighted contrast-enhanced (T1ce), T2, FLAIR, standard Diffusion-Weighted Imaging with vendor-provided Apparent Diffusion Coefficient, a multi-shell diffusion sequence known as RSI, Susceptibility-Weighted Imaging, DSC perfusion imaging, and ASL perfusion imaging with calibrated CBF maps from the vendor.

The imaging protocol included both pre- and post-gadolinium 3D volumetric T1-weighted inversion recovery spoiled gradient-recalled sequences (TE/TR = 2.8/6.5 ms, TI = 450 ms, flip angle = 8°, FOV = 24 cm, matrix = 0.93 × 0.93 × 1.2 mm) and a 3D T2-weighted FLAIR sequence (TE/TR = 126/6000 ms, TI = 1863 ms, FOV = 24 cm, matrix = 0.93 × 0.93 × 1.2 mm). For RSI, a single-shot pulsed-field gradient spin-echo echo-planar imaging (EPI) sequence was employed (TE/TR = 96 ms/17 s, FOV = 24 cm, matrix = 128 × 128 × 48, slice thickness = 2.5 mm), with 4 *b*-values (*b* = 0, 500, 1500, and 4000 s/mm^2^) and 6, 6, and 15 unique diffusion directions for each non-zero *b*-value, respectively, totaling 28 volumes and an approximate scan time of 8 min.

DSC perfusion MRI utilized a gradient-echo EPI sequence (TE/TR = 35/1600 ms, flip angle = 90°, slice thickness = 5 mm with a 1 mm inter-slice gap, 22 axial slices, FOV = 25 cm, matrix = 96 × 96). Contrast agents such as gadobenate dimeglumine (Bracco Diagnostics) or gadobutrol (Bayer AG) were injected intravenously at a dose of 0.1 mmol/kg using an MR-compatible power injector at a rate of 2-3 mL/s through an antecubital angiocatheter, followed by a 20 mL saline flush. Multi-section images were acquired every 1-2 s during the first pass of the contrast agent, with 60 timepoints collected.

All scans were performed on 3T clinical MRI scanners (GE Signa Excite HDx and GE Discovery MR750) using an eight-channel head coil at 2 different MRI facilities associated with UCSD. ASL perfusion MRI was conducted using pseudocontinuous (PCASL) labeling with a 3D stack-of-spirals fast spin-echo readout. Key PCASL parameters included a labeling duration of 1450 ms and a post-labeling delay of 2025 ms, with the 3D spiral readout utilizing 8 spiral interleaves, 512 points per spiral, 36 slices (slice thickness 4.0 mm), and a FOV of 24 cm. The in-plane resolution was 3.64 mm^2^, with a bandwidth of 62.5 kHz. Additional sequence parameters were TE = 10.5 ms, TR = 4847 ms, NEX = 3, and a total scan time of 4 min 42 s.

### Images Pre-Processing

The RSI images were processed using a standard pipeline outlined by White et al.^35^ to generate cellularity maps. The diffusion signal within each voxel was modeled using a linear mixture approach, distinguishing between restricted and hindered water compartments with spherical and cylindrical geometries. The spherically restricted water fraction served as the foundation for the RSI cellularity maps. To enhance sensitivity and specificity for this fraction, a beam-forming filter was applied to the cellularity maps, minimizing residual contamination from cylindrically restricted and hindered water compartments.

DSC images were processed using the Matlab DSC toolbox^36^ to calculate CBV, employing a leakage correction algorithm,^15^ as well as to compute CBF. For ASL-derived CBF maps, the 3D PCASL images were analyzed using ReadyView ASL (GE Healthcare). All imaging modalities were registered and resampled to align with a 256 × 256 × 256, 1 mm isotropic resolution MNI brain atlas.^37^ A brain mask was generated from the FLAIR images using a custom in-house nnUNet skull stripping network, which was subsequently applied to the other imaging modalities.

### Image Annotations

For each patient, cellular tumor segmentation was performed manually by an attending neuroradiologist (J.D.R.), with 3 years of post-fellowship experience and extensive segmentation expertise, alongside a neuroradiology fellow (L.G.). Segmentations were based on comprehensive clinical, pathological, and imaging history, including thorough reviews of prior and subsequent MRIs. Cases exhibiting post-treatment changes—such as PsP, radiation necrosis, and post-treatment changes alone—were further reviewed by a third neuroradiologist (N.F.) with 13 years of post-fellowship experience.

The neuroradiologists segmented the total cellular tumor (TCT), which included both enhancing and NECT regions. Enhancing cellular tumor (ECT) was derived by multiplying the reference standard segmentation with a conventional enhancing tumor (ET) mask generated using Deep Learning software (OnQ Neuro, Cortechs.ai, San Diego).^38^ The NECT mask was created by subtracting the ECT from the TCT. Additionally, OnQ Neuro segmented a conventional peritumoral infiltration and edema mask. While the tumor segmentation maps generated by OnQ Neuro were not manually modified, they were reviewed and approved by a neuroradiologist. We emphasize that NECT does not refer to treatment-related changes but rather infiltrative tumor. In the reference standard segmentations performed by the neuroradiologists, treatment-related changes were not included as cellular tumors. Segmentations of ECT and NECT were performed based on all available clinical, pathologic, and imaging history, including a thorough review of prior and subsequent MRI scans, which include the RSI cellularity maps. Enhancing post-treatment changes (such as radiation necrosis) were not included as ECT, and regions of FLAIR signal hyperintensity that did not show high cellularity on RSI maps were not included as NECT.

### Deep Learning Model

We employed a previously detailed auto-calibrating neural network, nnUNet.^39^ The post-processed images were fed into nnUNet using the standard configuration of the 3dfullres network. The reference standard TCT maps described earlier served as the target masks. Each model was trained for 1000 epochs, with training taking approximately 32 h per model on an NVIDIA RTX 3090 graphics card. The network was trained to segment TCT volumes, and the resulting mask was subsequently divided into estimated ECT and estimated NECT, following the method outlined above. A total of 5 different models were trained using various combinations of image inputs. Specifically, input combinations were: T1, T1ce, T2, FLAIR, RSI, ASL; T1, T1ce, T2, FLAIR, RSI, ASL-calibrated map (ASL-CBF); T1, T1ce, T2, FLAIR, RSI, DSC-CBV; T1, T1ce, T2, FLAIR, RSI, DSC-CBF; and T1, T1ce, T2, FLAIR, RSI (no perfusion).

### Performance Metrics

We assessed segmentation performance using the Dice score and volume similarity (VS). For multifocal disease, individual lesions were segmented separately, but global Dice and VS scores were calculated across all lesions combined. A 5-fold cross-validation analysis was conducted for all models. Twenty patients had repeated scans, and each scan was treated independently. To avoid any bias, we made sure that the repeated scans for those patients were used all together in either the training or the test set during the cross-validation, that is not used both in the training set and the test set. To evaluate each model’s ability to distinguish between residual or recurrent tumor and post-treatment changes, we calculated the area under the curve (AUC) of the receiver operating characteristic (ROC) curve. Cases with either progression or stability of residual disease were categorized as residual/recurrent, while those with PsP, radiation necrosis, or non-specific post-treatment changes were classified as post-treatment changes. For ROC construction, the independent variable was the TCT volume segmented by the model. The ROC curve was generated by varying the threshold of segmented TCT volume from 0 to 50 ml with increments of 0.1 ml, with cases exceeding the threshold classified as residual/recurrent and those below it considered post-treatment changes (negative). Comparison between AUCs produced by the different models was assessed with a *n* = 10 000 random permutation paired test to avoid making assumptions about the distribution of the data.^40^

## Results

### Segmentation Performance

**Figure 2.**
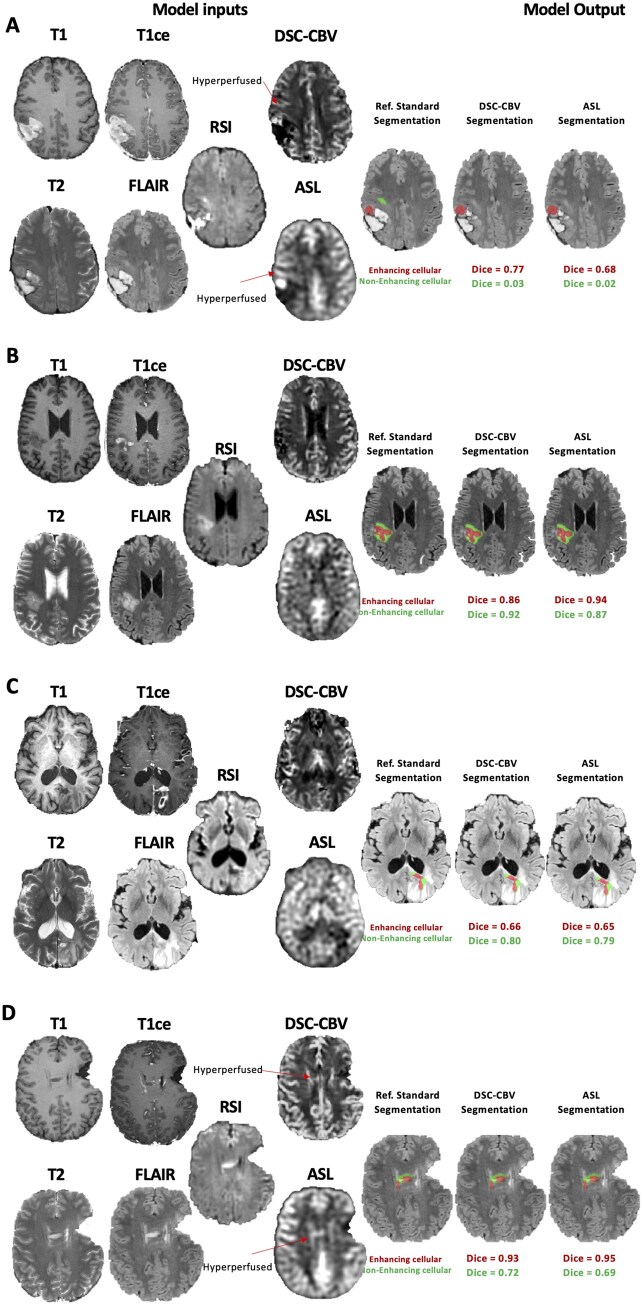
Examples of enhancing cellular tumor (red) and non-enhancing cellular tumor (green) segmentation output from the model trained with DSC-CBV and ASL (each panel A-D representing a distinct patient).

**Figure 3.**
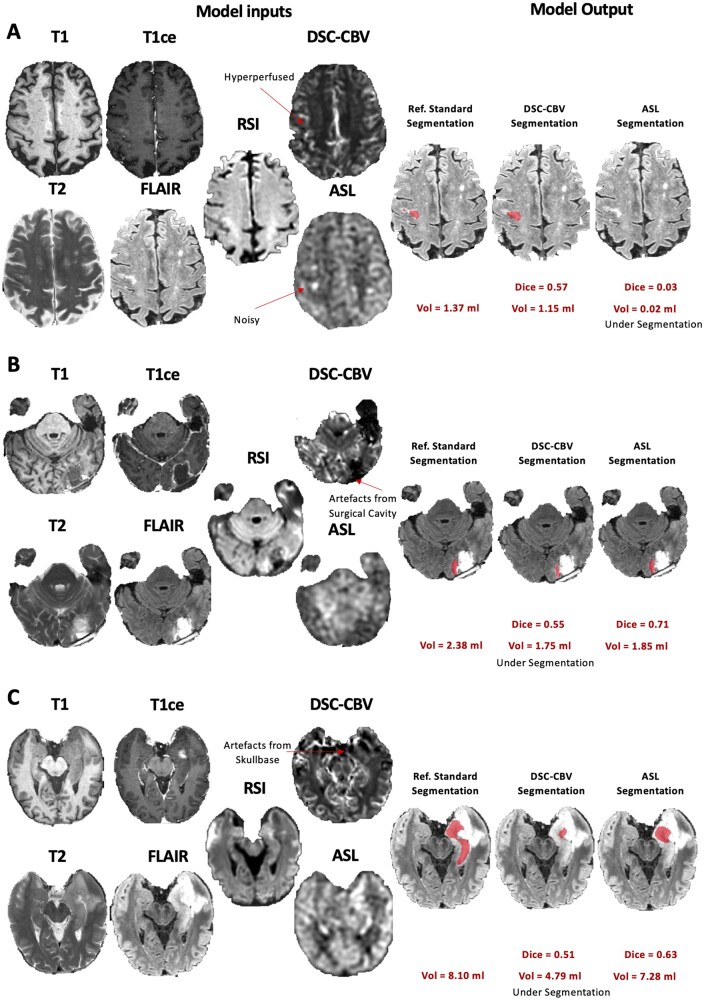
Cases for which the difference between the DSC-CBV and ASL model output resulted in a different clinical outcome (ie, recurrence detected vs missed) using a 1 mL cellular tumor volume threshold. (A) Case with noisy ASL map, resulting in missed recurrence with the ASL model but detected with the DSC-CBV model. (B) Case with susceptibility artifacts in the DSC map caused by the surgical cavity, resulting in undersegmentation and missed recurrence (subthreshold) with the DSC-CBV model but detected with the ASL model. (C) A case with susceptibility artifacts in the DSC map caused from proximity to the skull base, resulting in undersegmentation and missed recurrence (subthreshold) with the DSC-CBV model but detected with the ASL model.

The median volume of cellular tumor across the 138 timepoints in the reference standard segmentations performed by the neuroradiologist was 9.2 ml (IQR: 2.8-22.1) for TCT, 4.7 ml (IQR: 1.1-14.8) for ECT, and 2.2 ml (IQR: 0.7-5.6) for NECT. The median ratio of ECT/TCT volume was 0.66 (IQR: 0.38-0.86), and the median ratio of NECT/TCT volume was 0.34 (IQR: 0.14-0.62). Segmentation performance metrics computed for TCT, ECT, and NECT on the entire dataset (5-fold cross-validation, *n* = 138) are shown in Table 1. Dice scores obtained for TCT were similar (*P* = .93, paired *t*-test) for the model with ASL (median: 0.75, IQR: 0.53-0.84) and the model with DSC-CBV (median: 0.75, IQR: 0.57-0.84). Results were also similar between the model with ASL and DSC-CBV when comparing Dice scores for ECT (ASL: median: 0.84, IQR: 0.54-0.93; DSC-CBV: median: 0.85, IQR: 0.56-0.93; *P* = .62, paired *t*-test) and Dice scores for NECT (ASL: median: 0.49, IQR: 0.26-0.62; DSC-CBV: median: 0.47, IQR: 0.25-0.63; *P* = .85, paired *t*-test). Removing the perfusion (ie, model with T1, T1ce, T2, FLAIR, and RSI) did not decrease the overall segmentation performance (TCT: median: 0.76, IQR: 0.59-0.85, *P* = .53, paired *t*-test; ECT: median: 0.85, IQR: 0.54-0.93, *P* = .45, paired *t*-test; NECT: median: 0.49, IQR: 0.27-0.64, *P* = .70, paired *t*-test). Individual examples of segmentation outputs are shown in Figure 2. Cases where DSC-CBV performed better were mainly due to noisy ASL maps (eg, Figure 3A). Cases where ASL performed better included tumors located around surgical cavities and near the skull base, where DSC-CBV images contained artifacts (eg, Figure 3B and C). The model using the in-line calibrated ASL did not improve segmentation compared to the model using the raw subtracted ASL data (TCT: median: 0.76, IQR: 0.53-0.84 vs median: 0.75, IQR: 0.53-0.84, *P* = .13, paired *t*-test).

**Table 1.** Segmentation performance metrics^a^

	Dice		Volume similarity	
Model inputs	Mean ± SD	Median (25%-75% IQR)	Mean ± SD	Median (25%-75% IQR)
**Total cellular tumor**				
*T1, T1ce, T2, FLAIR, RSI*				
ASL	0.66 ± 0.24	0.75 (0.53-0.84)	0.78 ± 0.26	0.89 (0.67-0.95)
ASL-CBF	0.64 ± 0.26	0.76 (0.53-0.84)	0.75 ± 0.27	0.88 (0.63-0.95)
DSC-CBV	0.66 ± 0.25	0.76 (0.57-0.84)	0.78 ± 0.26	0.90 (0.73-0.95)
DSC-CBF	0.66 ± 0.26	0.76 (0.57-0.85)	0.78 ± 0.27	0.91 (0.70-0.95)
DSC-CBV & ASL	0.65 ± 0.26	0.75 (0.55-0.84)	0.78 ± 0.26	0.89 (0.73-0.95)
No Perfusion	0.67 ± 0.25	0.76 (0.59-0.85)	0.81 ± 0.25	0.92 (0.75-0.96)
**Enhancing cellular tumor**				
*T1, T1ce, T2, FLAIR, RSI*				
ASL	0.71 ± 0.29	0.84 (0.54-0.93)	0.78 ± 0.28	0.91 (0.70-0.97)
ASL-CBF	0.68 ± 0.31	0.83 (0.46-0.92)	0.75 ± 0.31	0.90 (0.65-0.97)
DSC-CBV	0.71 ± 0.28	0.85 (0.56-0.93)	0.78 ± 0.29	0.91 (0.66-0.97)
DSC-CBF	0.70 ± 0.30	0.84 (0.53-0.93)	0.78 ± 0.28	0.92 (0.69-0.97)
DSC-CBV & ASL	0.71 ± 0.30	0.83 (0.61-0.92)	0.78 ± 0.30	0.92 (0.74-0.97)
No Perfusion	0.72 ± 0.29	0.85 (0.54-0.93)	0.80 ± 0.28	0.93 (0.77-0.97)
**Non-enhancing cellular tumor**				
*T1, T1ce, T2, FLAIR, RSI*				
ASL	0.43 ± 0.26	0.49 (0.26-0.62)	0.64 ± 0.31	0.73 (0.47-0.87)
ASL-CBF	0.42 ± 0.26	0.47 (0.22-0.62)	0.64 ± 0.31	0.74 (0.48-0.89)
DSC-CBV	0.43 ± 0.24	0.47 (0.25-0.63)	0.65 ± 0.30	0.75 (0.50-0.88)
DSC-CBF	0.43 ± 0.26	0.49 (0.19-0.63)	0.66 ± 0.30	0.76 (0.54-0.91)
DSC-CBV & ASL	0.42 ± 0.25	0.43 (0.22-0.61)	0.64 ± 0.30	0.72 (0.46-0.87)
No Perfusion	0.44 ± 0.25	0.49 (0.27-0.64)	0.67 ± 0.29	0.76 (0.53-0.89)

Dice scores and volume similarities are shown as mean ± SD and median (interquartile range [IQR]).

Abbreviations: ASL, arterial spin labeling (raw substraction images); ASL-CBF, Calibrated Flow Map from ASL; DSC-CBF, dynamic susceptibility contrast computed cerebral blood flow; DSC-CBV, dynamic susceptibility contrast computed Cerebral Blood Volume; FLAIR, fluid-attenuated inversion recovery; RSI, Restricted Spectum Imaging Cellularity; T1, T1 pre-contrast; T1ce, T1 contrast-enhanced.

a No statistical differences were obtained between the 6 models using a paired *t*-test at the alpha = 0.05 level, for total cellular tumor, enhancing cellular tumor, and non-enhancing cellular tumor.

### Performance for Detecting Residual and Recurrent Tumor vs Post-Treatment Changes

**Figure 4.**
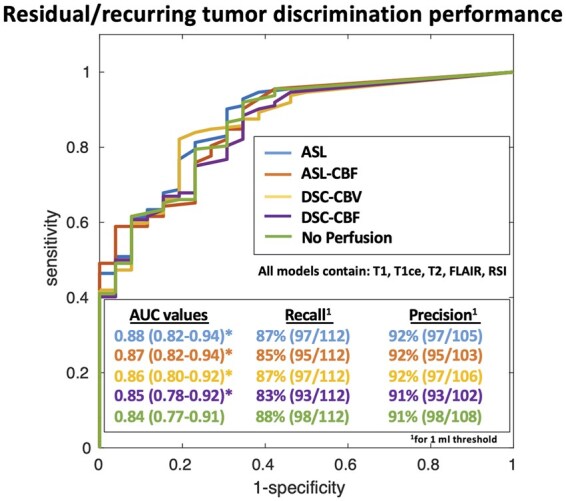
Receiver operating characteristic (ROC) curves illustrating the relative performance of ASL and DSC perfusion to discriminate residual/recurrent tumor from post-treatment changes. All models used T1, T1ce, T2, FLAIR, and RSI cellularity with either ASL or DSC perfusion or without perfusion. AUCs are indicated with 95% CI in parenthesis. *indicates statistical significance at the ⍺ = 0.05 level (*n* = 10 000 random permutation test) compared to No Perfusion. ^1^Using a 1 mL of tumor volume as threshold.

The ROC curves computed over the entire dataset (5-fold cross-validation, *n* = 138) for different models are shown in Figure 4. The AUC of the ROC was 0.88 (CI, 0.82-0.94) for the model with ASL and 0.86 (CI, 0.80-0.92) for the model with CBV (Figure 4). This difference was statistically significant (*P* < .05, *n* = 10 000 random permutation test). Removing the perfusion decreased the performance (AUC: 0.84; CI, 0.77-0.91), and this difference was statistically significant (*P* < .05, *n* = 10 000 random permutation test). The details of the clinically meaningful differences are shown in Table 2. There were 3 positive cases missed by ASL and not by DSC (either DSC-CBV or DSC-CBF). Two out of 3 cases contained a high level of noise in the ASL images, and the other case had the tumor located in the midbrain. Individual examples of segmentation outputs for these missed cases by ASL are shown in Figure 3A. Eleven recurring cases were missed by the DSC (either DSC-CBV or DSC-CBF) model and not by the ASL model. Eight of these 11 cases contained artifacts in the DSC data (5 from the surgical cavity, 2 from an Ommaya reservoir, and 1 from the skull base). Individual examples of segmentation outputs for these missed cases by DSC are shown in Figure 3B and C. The recurring cases missed without perfusion but detected with perfusion (either DSC or ASL) were small and hyperperfused tumors, with individual cases shown in Figure S1.

**Table 2.** Clinically meaningful differences

	Number of cases
**Missed without perfusion**	**3**
Small hyperperfused tumors	3
**Missed by ASL but detected by DSC**	**3**
Noisy ASL	2
Tumor in the midbrain	1
**Missed by DSC but detected by ASL**	**11**
Artifacts from surgical cavity	5
Artifacts from skull base	1
Artifacts from Ommaya reservoir	2

## Discussion

In our analysis, 8 positives cases were detected by the ASL model but missed by the model with DSC due to the presence of artifacts in the DSC data. In 5 out of 8 cases, the recurring tumor was located in the margins of the surgical cavity, which created susceptibility artifacts in the DSC images. This better performance of ASL over DSC for assessing perfusion in tumors on the margins of surgical cavities is consistent with a previous study (Manning et al.^32^) Similarly, 2 positive cases were erroneously called negative with the DSC model because of artifacts from an Ommaya reservoir. Finally, 1 positive case was missed by the DSC model because the recurring tumor was located close to the skull base, where susceptibility artifacts can be encountered in the DSC images. This result is also consistent with previous work.^32^ By being less sensitive to the value of the local magnetic field, ASL can avoid these artifacts and provide better perfusion assessment in those specific regions. In those 8 cases, the result was clinically significant since the outcome changed from negative to recurring disease. Our result emphasized that performing ASL perfusion measurements on top of traditional DSC perfusion measurements can ­positively change the clinical management if the data are ­analyzed with Deep Learning.

Our results showed that both ASL and DSC increased the detection of recurrent/residual disease compared to when no perfusion was used. This result is consistent with previous studies (reported in a meta-analysis by Taylor et al.^3^). This result re-emphasizes the need to include a perfusion measurement in a brain tumor protocol. As stated above, our results suggest that ASL performs as well as DSC in most cases and better in specific cases where DSC is contaminated by susceptibility artifacts. This result is also consistent with previous studies.^32^^,^^33^ The cases detected when including perfusion but missed when no perfusion was used were those in which the tumor was small and hyperperfused and therefore under-segmented when no perfusion was included. For small segmented tumor volume thresholds, the performance of the no-perfusion model to classify cases as recurring or not will be similar to other models that use perfusion. However, since the volume of the segmented tumor is smaller (see Figure S1A and B), by gradually increasing the threshold to produce the ROC curve, the no-perfusion model will miss these small hyperperfused tumor cases, which will result in a lower AUC. As for the segmentation performance, perfusion images are usually noisy, and the Dice score obtained using perfusion is usually not higher than the Dice score obtained without perfusion. In other terms, perfusion improves detection but not necessarily the precision of the segmentation.

Differentiating recurrent ETs from post-treatment changes in GBM patients is crucial for clinical management but remains challenging even for experienced clinicians. As in our previous work, the Deep Learning method presented here allowed to segment cellular tumor volumes, which can be used to distinguish recurrent/residual tumors from post-treatment changes. The optimal AUC under the ROC curve obtained here (0.88; CI, 0.82-0.94) is slightly higher compared to the one obtained in our previous work (0.84; CI, 0.79-0.89) since only a subpart of the dataset containing ASL images was used (*n* = 138 out of 243 cases).

Our results confirm that adding a perfusion measurement improves the detection of recurring GBM from Deep Learning segmentation of cellular tumors on post-operative multimodal MRI, and this improvement changed positively the clinical outcome in a few cases. While the overall performance of DSC and ASL is very similar, ASL performs better in specific instances where susceptibility artifacts can decrease the quality of DSC, including in the periphery of a surgical cavity with blood products, close to the skull base, and adjacent to an Ommaya reservoir. Our results emphasize the benefits of including ASL in brain tumor protocols.

### Limitations

In this work, the ASL images were processed in-line from the MRI scanner for both the raw subtracted images and the calibrated ASL-CBF perfusion map. While the calibrated ASL-CBF perfusion map did not improve segmentation performance, the un-subtracted ASL images were not available, and therefore no fine-tuning of the ASL processing could be performed. The influence of different ASL processing streams could be quantified in future work using datasets containing un-subtracted ASL images. Moreover, the performance of our method could be compared against other perfusion metrics (such as maxCBV) and conventional neuroradiologist assessment. A second limitation is the small size of our dataset. Since ASL is becoming more widely available, more datasets of brain tumor with ASL images will be available to increase the statistical power of our results. A third limitation is the lack of histological validation of our cellular tumor maps, which will be addressed in future work.

## Supplementary Material

vdaf219_Supplementary_Data

## Data Availability

The dataset used in this study can be found on The Cancer Imaging Archive (TCIA) website DOI: 10.7937/fwv2-dt74
