# Supplementary material for: Comparing the performance of dynamic susceptibility contrast and arterial spin labeling for detecting residual and recurrent glioblastoma with deep learning and multishell diffusion MRI
Source: Neurooncol Adv. 2025 Oct 17;7(1):vdaf219. doi: 10.1093/noajnl/vdaf219 (PMC12768508; doi:10.1093/noajnl/vdaf219)
Supplement: vdaf219_Supplementary_Data [file vdaf219_supplementary_data.zip › supplementary_table_1.docx]

**Supplementary Table 1.** Patient Demographics and Tumor statistics

|  | UCSD cohort  5-fold cross validation |
| --- | --- |
| **Demographics** |  |
| Timepoints | 138 |
| Patients | 107 |
| Age (years) | 57 ± 13 |
| Men | 70 (65%) |
| Women | 37 (35%) |
| GTR + | 54 (50%) |
| Recurring/residual | 112 (81%) |
| Negative | 26 (19%) |
