# Supplementary material for: Comparing the performance of dynamic susceptibility contrast and arterial spin labeling for detecting residual and recurrent glioblastoma with deep learning and multishell diffusion MRI
Source: Neurooncol Adv. 2025 Oct 17;7(1):vdaf219. doi: 10.1093/noajnl/vdaf219 (PMC12768508; doi:10.1093/noajnl/vdaf219)
Supplement: vdaf219_Supplementary_Data [file vdaf219_supplementary_data.zip › supplementary_table_and_figure_legends.docx]

**Supplementary Table legends**

**Supplementary Table 1.** Patient Demographics and Tumor statistics

**Supplementary Figure legends**

**Supplementary Figure 1.** Example of model output for recurring cases detected with perfusion but missed when the model did not included perfusion as an input. Those cases were small and hyper-perfused tumors
