# Supplementary figures and images for: Comparing the performance of dynamic susceptibility contrast and arterial spin labeling for detecting residual and recurrent glioblastoma with deep learning and multishell diffusion MRI
Source: Neurooncol Adv. 2025 Oct 17;7(1):vdaf219. doi: 10.1093/noajnl/vdaf219 (PMC12768508; doi:10.1093/noajnl/vdaf219)

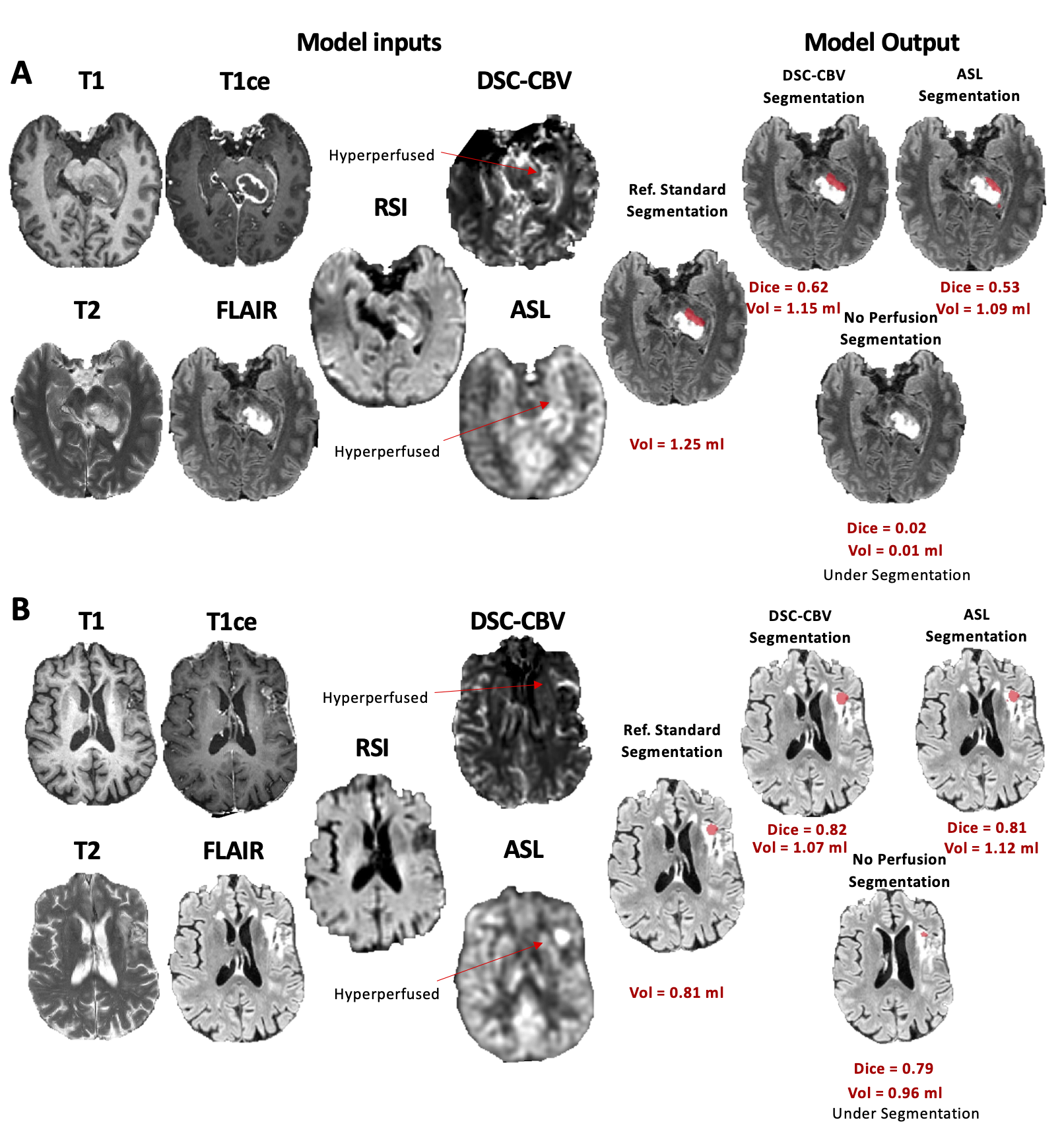

Supplement: vdaf219_Supplementary_Data [file vdaf219_supplementary_data.zip › supp_figure_1.tiff]
